# *In vitro* Effects of Four Native Brazilian Medicinal Plants in CYP3A4 mRNA Gene Expression, Glutathione Levels, and *P*-Glycoprotein Activity

**DOI:** 10.3389/fphar.2016.00265

**Published:** 2016-08-19

**Authors:** Andre L. D. A. Mazzari, Flora Milton, Samantha Frangos, Ana C. B. Carvalho, Dâmaris Silveira, Francisco de Assis Rocha Neves, Jose M. Prieto

**Affiliations:** ^1^Department of Pharmaceutical and Biological Chemistry, UCL School of PharmacyLondon, UK; ^2^Faculdade de Ciências da Saúde, Universidade de BrasíliaBrasília, Brazil; ^3^Agência Nacional de Vigilância Sanitária, Coordenação de Medicamentos Fitoterápicos e DinamizadosBrasília, Brazil

**Keywords:** herbal medicines, Brazil, glutathione, CYP3A4, *P*-Glycoprotein, drug metabolism

## Abstract

*Erythrina mulungu* Benth. (Fabaceae), *Cordia verbenacea* A. DC. (Boraginaceae), *Solanum paniculatum* L. (Solanaceae) and *Lippia sidoides* Cham. (Verbenaceae) are medicinal plant species native to Brazil shortlisted by the Brazilian National Health System for future clinical use. However, nothing is known about their effects in metabolic and transporter proteins, which could potentially lead to herb-drug interactions (HDI). In this work, we assess non-toxic concentrations (100 μg/mL) of the plant infusions for their *in vitro* ability to modulate CYP3A4 mRNA gene expression and intracellular glutathione levels in HepG2 cells, as well as *P*-glycoprotein (*P*-gp) activity in vincristine-resistant Caco-2 cells (Caco-2 VCR). Their mechanisms of action were further studied by measuring the activation of human pregnane X receptor (hPXR) in transiently co-transfected HeLa cells and the inhibition of γ-glutamyl transferase (GGT) in HepG2 cells. Our results show that *P*-gp activity was not affected in any case and that only *Solanum paniculatum* was able to significantly change CYP3A4 mRNA gene expression (twofold decrease, *p* < 0.05), this being correlated with an antagonist effect upon hPXR (EC50 = 0.38 mg/mL). Total intracellular glutathione levels were significantly depleted by exposure to *Solanum paniculatum* (-44%, *p* < 0.001), *Lippia sidoides* (-12%, *p* < 0.05) and *Cordia verbenacea* (-47%, *p* < 0.001). The latter plant extract was able to decrease GGT activity (-48%, *p* < 0.01). In conclusion, this preclinical study shows that the administration of some of these herbal medicines may be able to cause disturbances to metabolic mechanisms *in vitro*. Although *Erythrina mulungu* appears safe in our tests, active pharmacovigilance is recommended for the other three species, especially in the case of *Solanum paniculatum*.

## Introduction

According to the World Health Organization (WHO), 65–80% of the world’s population in developing countries depends on medicinal plants for their primary health care due to poverty or lack of access to modern medicine (Silveira et al., 2008). Latin American countries possess an enormous part of the world’s biodiversity, with Brazil alone containing approximately 22% of all existing plants and microorganisms on earth (Calixto, 2005). For the past 10 years, Brazilian health care authorities have directed their attention to the considerable use of medicinal plants in hope to integrate traditional Brazilian medicine into the public health care system (SUS). Indeed, there is a growing demand for the use of medicinal plants by the citizens of Brazil as well as an interest in natural products to support healthier lifestyles (Silveira et al., 2008).

Since 2004, the SUS, along with the Ministry of Health (MoH), have created a National Policy on Integrative and Complementary Practices (PNPIC), which is aimed at offering traditional medicinal plants as a treatment option through the SUS with guaranteed safety and quality (Balbino and Dias, 2010). The MoH and the SUS surveyed municipalities nationwide and, using indications according to categories of the International Classification of Diseases, created the National List of Medicinal Plants of Interest to SUS (RENISUS)^1^. This list is composed of 71 plants, which have the potential to generate products of interest for the SUS, as well as promote the traditional practice of herbal remedies. In the same year, the Brazilian Health Surveillance Agency (ANVISA) began to regulate the registration of herbal medicines, requiring quality control and safety reports confirming good manufacturing practice for all herbal medicines (Oliveira et al., 2006). As a result of this advancement, the first phytomedicine to be fully developed in Brazil was approved by ANVISA. It is an anti-inflammatory topical ointment marketed as Acheflan^®^, in which the active ingredient is the Brazilian medicinal plant *Cordia verbenacea* DC (Boraginaceae), a plant analyzed in this study (Calixto, 2005).

Plant species included in the RENISUS list are prioritized to undergo safety and efficacy studies. To date, very little is known about the effects they may cause on phase 1 and phase 2 metabolism and transporter proteins. Also, the pharmacokinetic (PK) profile of herbal medicines is virtually impossible to study as they are Complex Chemical Entities, i.e., consisting of numerous chemicals with disparate absorption rates, pharmacodynamic (PD) and PK properties (He et al., 2011). Therefore, the preclinical approach should clarify whether herbal medicines can alter the activity and expression of discrete metabolic and/or transporter proteins in order to theorize which prescription drugs (single chemical entities) would be affected by their concomitant use.

Since CYP3A4 metabolizes most of the currently marketed drugs, glutathione is a central player in drug conjugation and *P*-glycoprotein (*P*-gp) activity determines drug absorption, most of the preclinical safety studies of natural and synthetic drugs focus on these targets (Zhou, 2008; Glaeser, 2011; Lu, 2013).

Such data is necessary in order to predict and avoid interactions that may occur between the extracts and conventional drugs. These types of interactions are called herb-drug interactions (HDI) (Mazzari and Prieto, 2014b). In a previous literature review, we revealed that such data are known for only half of the medicinal plants traditionally used in Brazil (Mazzari and Prieto, 2014a). In order to fill this information gap, we embarked on a study of 24 medicinal plants for which no information of this sort was found. Here we report on our results for four native plants on RENISUS, namely *Erythrina mulungu* Benth. (Fabaceae), *Cordia verbenacea* A. DC. (Boraginaceae), *Solanum paniculatum* L. (Solanaceae) and *Lippia sidoides* Cham. (Verbenaceae), which are used in the treatment of symptoms ranging from anxiety to gastric dysfunctions (**Table 1**).

**Table 1:** Botanical, pharmacopoeial, pharmacological and phytochemical information of the four Brazilian native species.

Family	Scientific name	Popular name	Brazilian pharmacopeia edition	Part	Traditional indication and experimental use	Pharmaceutical formulation	Chemistry
Leguminosae	*Erythrina mulungu* Mart. Ex Benth	Mulungu/Murungu/Suina/Sapatinho de judeu/Bico de papagaio (Brandão et al., 2006)	1st (1926)/2nd (1959) (Brandão et al., 2006)	Wood (Brandão et al., 2006)	Mild sedative/Insomnia/Depression (Setti-Perdigão et al., 2013)	Fluid-extract/Tincture/Vegetal drug (Brandão et al., 2006)	Alkaloids (+)-11α-hydroxy-erythravine/Erythravine/(+)-α-hydroxyerysotrine (Flausino et al., 2007)
Solanaceae	*Solanum paniculatum L.*	Jurubeba/Juripeba/Jubeba/Juvena/Juúna (Brandão et al., 2006)	1st (1926) (Brandão et al., 2006)	Roots (Brandão et al., 2006)	Tonic/Anti-fever agent/Colagogue/Bitter/Eupeptic to treat liver and gastric dysfunctions (Mesia-Vela et al., 2002; Martins et al., 2015; Vieira Junior et al., 2015) Herpes Virus (HHV-1) (Valadares et al., 2009) Chemoprevention agent (Endringer et al., 2010)	Tincture/Dry Extract/Fluid-Extract/Vegetal Drug (Brandão et al., 2006)	Flavonoids (Matias et al., 2016) Alkaloids (Jurubebina, Jubebine, Solanine) Resins (Jupebina, Jupebenina) Saponines (Isojuripidine, Isojurubidine, Isopaniculidine and Jurubidine) (Mesia-Vela et al., 2002)
Verbenaceae	*Lippia sidoides* Cham.	Alecrim-pimenta (ANVISA, 2011)^∗^	Formulário de Fitoterápicos da Farmacopéia Brasileira – 1^a^ Edição – 2011 (ANVISA, 2011)^∗^	Leaves (ANVISA, 2011)^∗^	Anti-septic/Anti-microbial (Almeida et al., 2010)	Extemporaneous preparations/Tincture/Gel/Soap (ANVISA, 2011)^∗^	Flavonoids/Quinones/Triterpenes/Lignanes/Free and glycosylated steroids/Organic acids (Almeida et al., 2010)
Boraginaceae	*Cordia verbenaceae* DC.	Erva – baleeira (ANVISA, 2011)^∗^	Formulário de Fitoterápicos da Farmacopéia Brasileira – 1^a^ Edição – 2011 (ANVISA, 2011)^∗^	Leaves (Ventrella and Marinho, 2008; ANVISA, 2011)^∗^	Anti-inflammatory/Analgesic/Anti-ulcerogenic/healing agent (Ventrella and Marinho, 2008)	Tea/Infusions (Ventrella and Marinho, 2008)	Monoterpenes/Sesquiterpenes/Triterpenes/Flavones (Ventrella and Marinho, 2008)

*^∗^ANVISA, A.N.D.V.S. (2011). *Formulário de Fitoterápicos – Farmacopéia Brasileira, Brasilia*. Available at: http://www.anvisa.gov.br/hotsite/farmacopeiabrasileira/conteudo/Formulario_de_Fitoterapicos_da_Farmacopeia_Brasileira.pdf [accessed June 16, 2013].*

## Materials and Methods

### Chemicals and Reagents

#### Cell Culture

HepG2 and Caco-2 cell lines were from Sigma Aldrich at passages 100 and 43 respectively. HeLa cells were donated by Dr. Paul Webb from the Houston Methodist Institute for Technology at passage 20. All cell culture reagents were from Gibco^®^ Invitrogen unless otherwise stated. Vincristine 2 mg/mL was purchased from Hospira Ltd.

#### HPTLC Analysis

Water, Dichloromethane (>99.8%, contains amylene as a stabilizer), ethyl acetate (>99.7%) and methanol (>99.9%) all ChromasolvPlus for HPLC provided by Sigma Aldrich. Acetic acid (glacial) analytical reagent grade provided by Fisher Scientific. Formic Acid 98% provided by Rectapur^®^ VWR.

Caffeic acid was from Kotch-light Laboratories LTD. Rutin hydrate, quercetin dehydrate and gallic acid all from Sigma Aldrich. Diphenylborinic acid 2-aminoethyl ester (98%) and coumarin laser grade (99% UV-Vis) were provided by ACROS organics. Luteolin (HPLC grade) was from Extrasynthése. Chlorogenic acid provided by Cayman Chemical Co. Polyethylene glycol 4000 grade was from Fisher Scientific.

#### Cell Viability Assays

Sulphorhodamine B (SRB), Neutral Red (NR), (3-[4,5-dimethylthiazol-2-yl]-2,5 diphenyl tetrazolium bromide) (MTT), trichloroacetic acid (TCA), glacial acetic acid, 96% ethanol, hydrochloric acid (HCl), Isopropanol and Tris base were from Sigma Aldrich.

#### CYP3A4 mRNA Gene Expression Assay

Oligonucleotide primers were custom-synthesized by Invitrogen Life Technologies and Sigma Aldrich. TRIzol^®^ (Total RNA Isolation Reagent), Oligo (dT) 12-18 primers, M-MLV Reverse Transcriptase, RNAseOUT, DNAse I Amplification Grade and 100 mM dNTP Set were purchased from Invitrogen Life Technologies. SYBR Green (2xqPCR Master Mix premixed with SYBRgreen) was obtained from Amethyst reagents (Cambridge Bioscience). Rifampicin and DMSO were purchased from Sigma Aldrich.

#### hPXR Assay

Lipofectamine^®^ 2000 was purchased from Invitrogen. Rifampicin and Luciferase assay system were obtained from Sigma Aldrich and Promega, respectively.

#### Intracellular Glutathione Assay

Buthionine sulfoximine (BSO), L-glutathione reduced, glutathione reductase, 5-5′-dithiobis(2-nitrobenzoic acid) (DTNB), β-Nicotinamide adenine dinucleotide 2′-phosphate reduced tetrasodium salt hydrate (NADPH), Triton-X and sulfosalicylic acid were from Sigma Aldrich.

#### GGT Activity Assay

L-Glutamic acid γ-(p-nitroanilide) hydrochloride, 4-nitroaniline, glycyl-glycine (Gly-Gly), Tris base and acivicin were from Sigma Aldrich.

#### Rhodamine 123 Uptake Assay

Rhodamine 123 was from Sigma Aldrich. Verapamil (Securon IV 2.5 mg/mL) was from Abbott Laboratories Ltd.

### Plant Materials and Extraction

Plant materials were collected from “Farmácia Viva Brasília” (Brasília, DF – Brazil); via the University of Brasília – UnB. The plants were grown and processed according to good practices, ensuring validity and quality^2^.

All whole plant handling and extraction were performed at UnB. Aerial parts of the plants (100 g) were subjected to a 20 min infusion, to mimic traditional use. The infusions were filtered, lyophilized, and immediately sent to UCL School of Pharmacy. The extracts were stored at -18°C throughout the studies.

Special permission must be granted from the Brazilian Council for Management of Genetic Heritage (CGEN) in order to have access to genetic material, respecting international intellectual and genetic property rights laws. Access was authorized (license n. 010295/2014-3) resulting from collaboration between University College London (UCL) and UnB. Access was granted on May 2014, and the plant extracts were received on June 2014.

### HPTLC Analysis

Extracts were diluted to a concentration of 50 mg/mL in methanol. Control compounds were made at a concentration of 1mg/mL, also diluted in methanol. A CAMAG Linomat 5 was used to apply 5 μL of the samples to TLC silica gel 60 F254 aluminum sheets. The plates were developed using a CAMAG ADC2 automatic developing chamber. The method included 30-s pre-drying, 10min humidity control using magnesium chloride to 48.3% relative humidity and 20 min saturation time, using saturation pads all done at 25.2°C. The mobile phase used was ethyl acetate: formic acid: water (82:9:9). During development, the solvent front was allowed to migrate 80 mm before a drying time of 5 min. For derivatization, we used Natural products reagent (NPR) followed by PEG 4000 (Reich and Schibli, 2007). All visualization and analysis were done using CAMAG TLC visualizer both before and after derivatization.

### Cells Culture

HepG2 cells were cultured in Minimal Essential Medium (MEM) Alpha supplemented with 10% fetal bovine serum (FBS), 100 U/mL penicillin, 100 μg/mL streptomycin. Caco-2 cells were cultured in Dulbecco’s Minimum Essential Medium (DMEM) with high glucose (4.5g/L) and L-glutamine supplemented with 10%FBS, 100 U/mL penicillin, 100 μg/mL streptomycin, 1% non-essential amino acids (NEEA) and Vincristine (50 μM). HeLa cells were cultured in DMEM media supplemented with 10% FBS, 100 U/mL penicillin, 100 μg/mL streptomycin. All cell lines were kept in the NuAire DH Autoflow CO_2_ Air-Jacketed incubator at 37°C/5%CO_2_.

### Cell Viability Assays

The SRB, NR and MTT assays were performed as previously described (José Ruiz et al., 2006; Houghton et al., 2007; Repetto et al., 2008).

### Real-Time RT-qPCR Analysis

#### mRNA Extraction and cDNA Synthesis

After exposing HepG2 cells (5 × 10^5^ cells/well) to plant extracts or the CYP3A4 inducer Rifampicin (50 μM) or the CYP 3A4 inhibitor DMSO 1% for 96 h, total RNA was extracted from using TRIzol^®^ Reagent according to the manufacturer’s protocol. Samples were treated with DNase I (1 U/μL) to avoid genomic contamination. The quantity and quality of RNA was determined by differential readings at 260 and 280 nm in a Nanodrop 2000 (Thermo Scientific). The integrity of total RNA from HepG2 cells was assessed by visual inspection of the two rRNAs 28 and 18 s on agarose gels. cDNA was synthesized from 1 μg of total RNA with the Moloney rine Leukemia Virus Reverse Transcriptase (M-MLV RT) (200 U/μL) and oligo(dT) 12-18 primer (0.5 μg/μL), according to the manufacturer’s instruction in a final volume of 21 μL.

#### RT-qPCR Conditions and Analysis

CYP3A4 sense strand primer sequence was 5′-CAAGGACAACATAGATCGTTACATATACACACCCTTTGGAAG-3′ and the antisense strand primer was 5′-AGCTCAATGCATGTACAGAATCCCCGGTTA-3′ (Usui et al., 2003). The β-actin gene was used to control for variations in RNA loading within the experimental conditions. The sense strand primer sequence was 5′-CGTACCACTGGCATCGTGAT-3′ and the antisense strand primer was 5′-GTGTTGGCGTACAGGTCTTTG-3′. The RT-qPCR was carried out in 96-well plates using a Pikoreal^TM^ Real-Time PCR detection system (Thermo Scientific). Each well contained a final reaction volume of 10 μL (5.0 μL MasterMix with SYBR Green, 2.0 μL cDNA template diluted appropriately, 0.5 μL of each primer at a final concentration 0.3 mM and 2.0 μL of RNAse/DNAse free distilled water). PCR reaction was performed under the following schema: initial denaturation at 95°C for 2 min, then 40 cycles of denaturation at 95°C for 15 s, annealing at 55°C (β-actin) or 60°C (CYP3A4) for 30 s, and extension at 72°C for 30 s.

At the end of the run, a melting curve was generated by heating the amplicon from 60 to 95°C in order to confirm the specificity of the amplification for each primer pair. All RT-qPCR were run in quadruplicates. Standard curves were produced to check the PCR efficiency using a fivefold dilution series of cDNA. Efficiency (E) of primer pairs was obtained from the slope of the calibration curve generated. The relative expression was calculated on the basis of ‘delta delta Ct’ (ΔΔCt) values. Normalization of the target gene was achieved by using β-actin as a reference gene.

### hPXR Activation Assay

After 24 h seeding, HeLa cells (4 × 10^4^ cells/well) were transiently co-transfected with 60 ng of pM-Gal4-PXR-LBD and 240 ng of Gal4 luciferase reporter using lipofectamine 2000 reagent according to the manufacturer’s protocol. Transfected cells were treated with increasing concentrations of plant extract and/or rifampicin 1 μM (EC50). Luciferase activity was measured after 24 h, according to manufacturer’s protocol in a 20/20 n Glomax luminometer and reported as a response (%) compared to cells treated only with rifampicin.

### Intracellular Glutathione Levels

The method used in the intracellular determination of glutathione levels was adapted from those described by Allen et al. (2000) and Rahman et al. (2006) with slight modifications. After 24 h incubation with BSO (10 μM) or plant extracts (100 μg/mL), HepG2 cells (4 × 10^4^ cells/well) were washed with PBS and 60 μL of 0.1% Triton-X was added to each well of the plates to lyse the cells. Twenty five micro liter of 5% sulfosalicylic acid was added to the cell lysates and plates were shaken for 2 min. Twenty five micro liter of glutathione reaction buffer containing NADPH (2.39 mM), DTNB (0.01 M) and glutathione reductase (500UI) in sodium phosphate buffer (143 mM) containing EDTA (6.3 mM) was added to the cell lysates. Absorbance was read in a kinetic cycle in the plate reader every 30 s for 5 min at 405 nm (11 readings). Absorbances were converted into absolute amounts by means of the i-slopes method using known concentrations of L-glutathione reduced.

### GGT Activity Assay

GGT activity assay was conducted according to Rebbeor et al. (1998) with slight modifications. Briefly, after 24 h incubation of HepG2 cells (1 × 10^6^ cells/well) with the GGT inhibitor acivicin (5 μM) or plant extracts (100 μg/mL), media was aspirated and cells were washed with PBS. Four milliliter of pre-warmed glycylglicine buffer (115 mM Tris, 138mM glycylglycine) and 400 μL of the substrate γ-glutamyl-p-nitroanilide (29.6 mg/mL of HCl 0.5 mmol/L) were added to the wells and plates were incubated for 10 min. Then, 500 μL of the content of each well were transferred to 24-well plates and absorbance was measured in the plate reader (405 nm). Absorbances were converted into absolute amounts by means of a calibration line using 4-nitroaniline.

### Rhodamine 123 Uptake Assay

Rhodamine uptake/eﬄux assays were conducted as described by Chieli et al. (1993) with minor modifications. After five passages in media containing vincristine (50 μM), Caco-2 VCR (Eneroth et al., 2001) cells (1 × 10^4^ cells/well) were incubated for 2 h with the *P*-gp inhibitor verapamil (20 μM) or plant extracts (100 μg/mL) in serum-free media containing rhodamine 123 (5 μg/mL). After incubation, cells were washed with verapamil (20 μM) in PBS. Cells were lysed with 100 μL of 0.1% Triton X-100 in PBS and the plates were placed in the incubator for 15 min. The fluorescence intensity of cell lysates was measured in the plate reader (Exc-485 nm, Em-525 nm). The cellular accumulation of rhodamine 123 for each of the extracts was expressed as the percentage of the accumulation measured for rhodamine 123 under control conditions.

### Statistical Analysis

Collected data were analyzed as means ± SD of at least three independent experiments. Statistical significance was measured by student *t*-test and ANOVA followed by Bonferroni correction using GraphPad InStat (GraphPad Software Inc., La Jolla, CA, USA). Results with a value of *p* < 0.05 were considered significant.

## Results

### Yield of Plant Extracts

Yields of herbal extracts were as follows: *E. mulungu* (5.77%), *Solanum paniculatum* (9.27%), *Lippia sidoides* (13.33%) and *Cordia verbenaceae* (14.95%).

### HPTLC Analysis

*Cordia verbenaceae* fingerprint contains caffeic acid at retention factor (Rf) = 0.87 and chlorogenic acid at Rf = 0.38. *Solanum paniculatum* contains gallic acid at Rf = 0.81, rutin at Rf = 0.20 and chlorogenic acid at Rf = 0.38. *Lippia sidoides* contains luteolin at Rf = 0.86 and minor amounts of quercetin at Rf = 0.90. *E. mulungu* did not contain any of these metabolites in significant amounts (see Figure in Supplementary Material).

### Cell Viability

HepG2, Caco-2VCR, and HeLa cells exhibited more than 80% viability after 24 h incubation of 100 μg/mL of plant extracts. This allowed us to work on all experiments at that non-toxic concentration (Data presented as Supplementary Materials).

### Real-Time qPCR Efficiency

Both CYP3A4 and β-actin primers sequences revealed the specificity of target amplification. Baseline and threshold were properly set. Standard curve demonstrated good regression coefficient and efficiency (Data presented as Supplementary Materials). Melting curve analysis revealed a single peak for each pair of primers (Data presented as Supplementary Materials).

### CYP3A4 mRNA Gene Expression

**FIGURE 1:**
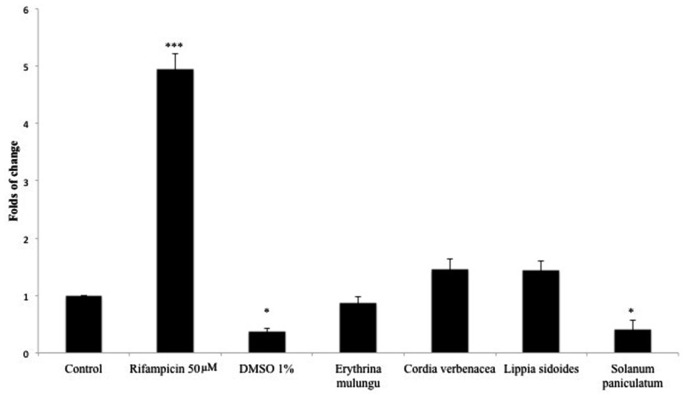
**CYP3A4 mRNA gene expression in HepG2 cells treated with rifampicin (50 μM), DMSO 1% and plant extracts (100 μg/mL).** After 4 days incubation with xenobiotics, CYP3A4 gene expression was determined as described in Section Real-Time RT-qPCR Analysis. Data are means ± SD; *n* = 4 experiments. ^∗^*P* < 0.05, ^∗∗∗^*P* < 0.001.

Rifampicin (50 μM), a known CYP3A4 inducer, was able to significantly increase CYP3A4 expression in 4.95 folds (*p* < 0.001). DMSO 1% inhibited CYP3A4 expression in 2.7 folds (*p* < 0.01). *E. mulungu, Cordia verbenacea* and *Lippia sidoides* were not able to modulate CYP3A4 expression in a significant manner compared to non-treated cells. However, *Solanum paniculatum* inhibited CYP3A4 mRNA gene expression in 2.4 folds, showing a similar effect to DMSO 1% (*p* < 0.01) (**Figure 1**).

### hPXR Antagonistic Effect of *Solanum paniculatum*

**FIGURE 2:**
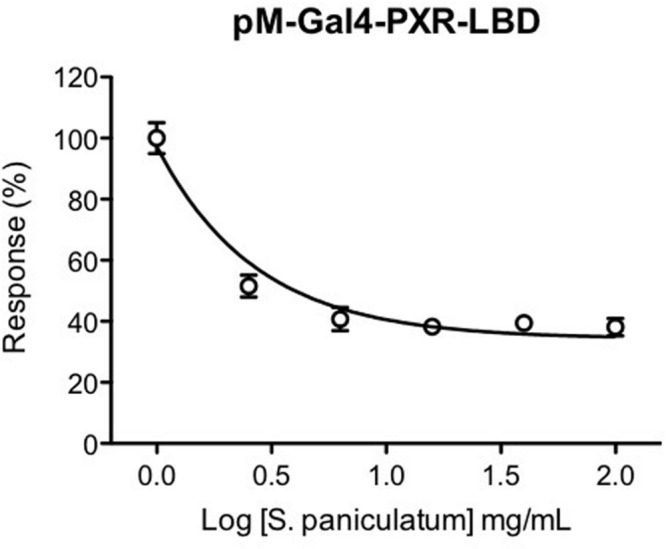
**Antagonistic effect of *Solanum paniculatum* extract on rifampicin-induced hPXR activation.** HeLa cells were co-transfected with expression vector pM-Gal4-PXR-LBD and Gal4 luciferase reporter and treated with increasing concentrations of *Solanum paniculatum* in the presence of rifampicin 1 μM (EC50). Luciferase activity was measured after 24 h and normalized as a percentage of rifampicin treated cells.

To further investigate whether the diminished expression of CYP3A4 mRNA gene by *Solanum paniculatum* treatment was mediated by an antagonist effect upon hPXR, we performed a reporter gene assay. As we can observe, co-transfected HeLa cells treated with rifampicin (1 μM) and serial dilutions of *Solanum paniculatum* extract showed a dose-response inhibition upon hPXR transcription activity. The maximal inhibition was close to 60% with a IC50 of 0.38 mg/mL (**Figure 2**).

Inhibition of hPXR reporter gene assay was not due to interference of the extract with the luciferase activity, since it did not show any effect on luciferase activity in HeLa cells transfected with CMV-luciferase expression vector. Additionally, this extract showed an agonist effect in HeLa cells co-transfected with thyroid hormone receptor beta 1 ligand binding domain and Gal4 luciferase reporter treated with triiodothyronine (T3). We did not use *Renilla* luciferase assay as an internal control since several authors have been describing the limitations of *Renilla* luciferase as an internal control of transcription efficiency (Ho and Strauss, 2004; Shifera and Hardin, 2010). This information is presented as Supplementary Data.

We also evaluated the effect of the other extracts on hPXR transcription activity. We observed that *E. mulungu* is a hPXR partial agonist since it increased hPXR reporter gene transcription activation in a dose dependent manner, but not as strong as rifampicin; *Lippia sidoides* did not show any effect on hPXR; *Cordia verbenaceae* failed to increase hPXR transcription activity. When we treated the cells with *Cordia verbenaceae* extract the transcription activity of rifampicin was impaired by 50% suggesting an antagonist effect. However, this is due to unspecific effects, since inhibition was also observed with a high affinity thyroid hormone receptor (Supplementary Figure S16, Supplementary Data).

### Modulation of Intracellular Glutathione Levels by Plant Extracts

**FIGURE 3:**
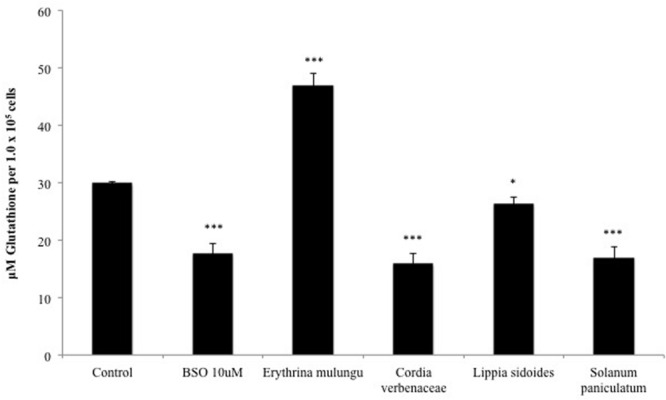
**Intracellular glutathione levels after 24 h treatment with the plant extracts (100 μg/mL) and buthionine sulfoximine (10 μM) in HepG2 cells.** Data are means ± SD; *n* = 4 experiments. ^∗^*P* < 0.05, ^∗∗∗^*P* < 0.001.

As shown in **Figure 3**, *Solanum paniculatum, Lippia sidoides* and *Cordia verbenacea* led to significant reduction of intracellular glutathione levels. The declines seen in the cells treated with *Solanum paniculatum* and *Cordia verbenacea* are statistically comparable to BSO (10 μM) (*p* < 0.001). *Lippia sidoides* also significantly inhibited the accumulation of glutathione in cells (*p* < 0.05). *E. mulungu*, on the other hand, was able to significantly increase the intracellular glutathione level (*p* < 0.001).

### GGT Activity Is Compromised by *Cordia verbenaceae*

**FIGURE 4:**
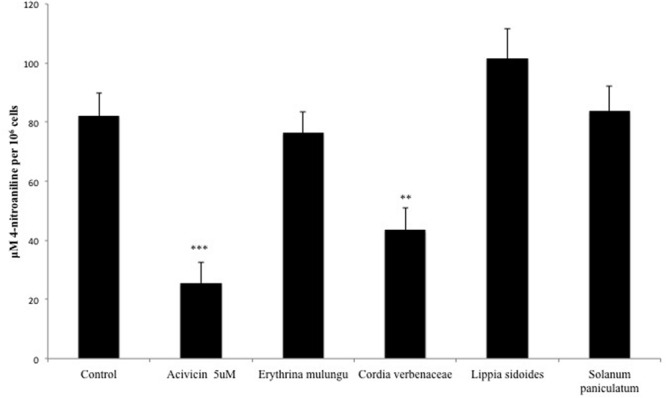
**γ-Glutamyl transferase (GGT) activity in HepG2 cells treated with acivicin (5 μM) or plant extracts (100 μg/mL).** GGT activity was measured on the surface of plated cells and the absorbances were converted into absolute amounts by means of a calibration line using 4-nitroaniline. Values are mean ± SD (*n* = 4). ^∗∗^*P* < 0.01, ^∗∗∗^*P* < 0.001.

The GGT activity in HepG2 cells was significantly lowered by *Cordia verbenacea* (*p* < 0.01). The GGT inhibitor acivicin was able to reduce its activity in a concentration of 5 μM (*p* < 0.001) (**Figure 4**).

### P-gp Eﬄux Activity Is Not Affected by the Plant Extracts

**FIGURE 5:**
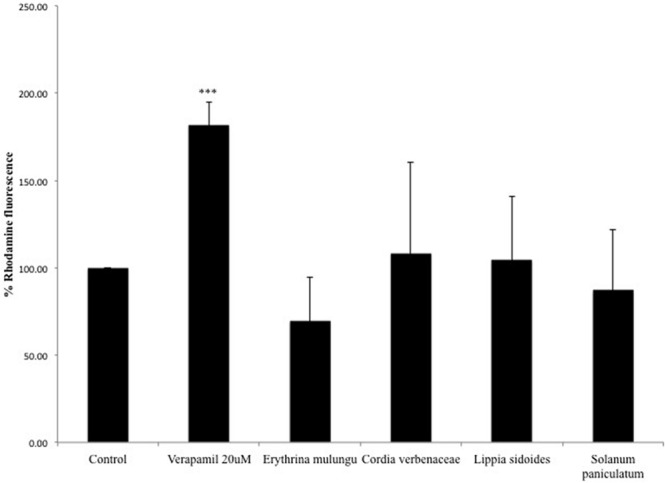
**Rhodamine 123 fluorescence in vincristine resistant Caco-2 cells (Caco-2 VCR).** Caco-2 VCR cells were treated with the *P*-gp inhibitor verapamil (20 μM) or plant extracts (100 μg/mL). Subsequently, cells were incubated with 5 μg/mL of rhodamine 123. Values are mean ± SD (*n* = 4).

Overexpression of *P*-gp protein and the eﬄux activity in Caco-2 VCR cells are confirmed by the results previously reported by Eneroth et al. (2001) and by experiment conducted in our lab (Data presented as Supplementary Materials). None of the plant extracts could significantly modulate *P*-gp activity in Caco-2 VCR cells at the tested concentration. The *P*-gp inhibitor verapamil (20 μM) was able to significantly impair the eﬄux of rhodamine 123 (*p* < 0.001) (**Figure 5**).

## Discussion

In Brazil, information about potential interactions caused by herbal medicines and conventional medicines alike, must be clearly described in the patient information leaflet. Besides HDI, other types of interactions should also be disclosed to the patient, such as drug-food, drug-chemical substance, drug-laboratory and non-laboratory test, and drug-disease interactions. Such data is available for newly registered herbal medicines, which have undergone preclinical and clinical tests. On the other hand, similar studies are not yet available for herbal medicines that are included in the ANVISA’s traditional herbal medicine list. In this case, the regulatory approval from ANVISA is given without the need of those studies.

To help in this endeavor, we investigate whether the four selected Brazilian native medicinal plants can alter the activity and/or expression of discrete metabolic and/or transporter proteins in order to evaluate which prescription drugs (single chemical entities) would be affected by their concomitant use.

*Solanum paniculatum* was the only medicinal herbal drug that decreased CYP3A4 gene expression. We show that this down-regulation is due –at least in part- to an antagonistic effect of the plant extract on hPXR. CYP3A is the most relevant family of phase 1 metabolism and CYP3A4 is responsible for metabolizing more than 50% of marketed drugs (Wienkers and Heath, 2005). hPXR is a nuclear receptor that plays a key role in the regulation of xenobiotic-inducible CYP3A mRNA gene expression and most cases of CYP3A4 gene regulation are related to the modulation of hPXR (Dong et al., 2010).

Our results must be put into the context of the limitations of HepG2 as a model for induction studies (Gerets et al., 2012). Although less sensitive than human hepatocytes (HH), HepG2 line is able to express CYP3A4 mRNA under the conditions established by Sumida et al. (2000). The poor response to induction is overcome by the sensitivity of RT-PCR, and this technique has been used for the study of natural products by several authors such as Cui et al. (2014) and Kumagai et al. (2016). Studies with HH should follow our screening to confirm its potential clinical significance (Sumida et al., 2000; Gerets et al., 2012; Cui et al., 2014; Kumagai et al., 2016). This could be the reason that we can see some antagonistic effects for *E. mulungu* in transfected HeLa cells but no modulation of CYP3A4 gene expression in HepG2 cells (See Supplementary Data).

*Solanum paniculatum*, together with *Cordia verbenacea* and *Lippia sidoides*, was also able to decrease intracellular glutathione levels, an important metabolite involved in the clearance of xenobiotics and the detoxification of reactive species (Xie, 2009). The variations of intracellular glutathione may be related to different effects, i.e., (a) inhibition/induction of enzymes involved in its biosynthesis, such as glutamate-cysteine ligase (GCL) and glutathione synthase (Lu, 2013); (b) the increase (or the inhibition) of GSH eﬄux from cells (Aw et al., 1986; Fernandez-Checa et al., 1988; Lu et al., 1990) which may be associated also with cell death (De Nicola and Ghibelli, 2014); (c) the formation (and the possible release) of GSH-adducts could also cause a reduction of the detectable intracellular glutathione (if it is not compensated by a new GSH synthesis) (Blair, 2010); finally (d) a modulation of glutathione transferase (GST) activity could also explain changes in both intra- and extracellular glutathione levels (Tolson and Wang, 2010). As for (a), the presence of active compounds in *Solanum paniculatum, Cordia verbenacea* and *Lippia sidoides* could be depleting glutathione in a similar manner BSO does, i.e., by inhibiting the GCL enzyme (Marengo et al., 2008). In order to identify these active compounds, a bioguided isolation strategy would be needed. Literature data reports that polyphenols such as gallic acid and derivatives, flavonoids such as luteolin, chrysin and apigenin, among others, induce GCL expression rather than inhibiting its activity (Panich et al., 2012; Huang et al., 2013). (b) is less likely to happen due to cell death as we are working with concentrations of plant extracts that are more than ten times lower than their maximum non-toxic concentration (>1000 μg/mL). More refined experiments would be needed to attest the formation of GSH-adducts, as stated in (c). As per (d) is another target that should be studied if the mechanism of glutathione depletion needs to be unveiled.

Depletion could be also linked to a non-functional γ-glutamyl cycle. This cycle serves as a continuous source of cysteine, which plays a key role in glutathione synthesis. GGT is an enzyme that first catalyzes the cleavage of glutathione on its γ-glutamyl-cysteine bond. Cysteinylglycine is then broken down by a dipeptidase to form glycine and cysteine. Cells reuptake those amino acids in order to synthesize more glutathione. Deficiency of GGT activity could potentially lower glutathione levels due to loss of cysteine (as glutathione) in the urine. Therefore, glutathione synthesis would be impaired due to the absence of this amino acid (Chevez-Barrios et al., 2000; Lu, 2013).

Indeed, the reduction of glutathione levels found in HepG2 cells treated with *Cordia verbenacea* turned out to be due –at least in part- by its ability to decrease GGT activity. However, *Solanum paniculatum* and *Lippia sidoides* did not affect GGT activity in a significant way, so glutathione levels in those cases could have been affected via other mechanisms.

Dereplication of the active principles responsible for this activity can be attempted from our HPTLC analysis. The flavonoid luteolin was found in *Lippia sidoides* and gallic acid was found in *Solanum paniculatum*. Those compounds have been reported to deplete intracellular glutathione levels. A study published by Balyan et al. (2015) demonstrated that intracellular glutathione levels were significantly depleted by luteolin in the human melanoma SK-MEL-28 cell line. This was explained by an inhibition of glutathione *S*-transferase (GST) activity via competitive reversible and irreversible mixed mechanisms (Balyan et al., 2015). Another study published by Locatelli et al. (2009) showed that gallic acid was able to deplete intracellular glutathione levels in melanoma cells through inhibition of the activity of γ-Glutamyl-cysteine synthetase. Gallic acid has been also proven to inhibit GGT activity in mice (Mahajan and Mahmood, 2009).

Last but not least, *P*-gp activity was not affected by any of the extracts. This is a positive finding taking into consideration that this is one of the most important targets in preclinical studies.

As for *E. mulungu*, it did not affect any of the targets selected in this work, however, it increased glutathione levels. Although this effect is not harmful in itself, its ability to cause HDIs cannot be ruled out by helping to metabolize other drugs through the formation of GSH adducts. We should still warn that other types of interactions may be important in this case. In Brazil, this medicinal plant has been used for a long time as a natural sedative. Indeed there is *in vivo* data validating its diazepam-like activity, so caution is warranted when patients have to combine this with other central nervous system (CNS) active prescription medicines, drive or operate potentially dangerous machines at work (Onusic et al., 2002).

Brazilian regulations on herbal medicines have been changing over the years. Improving the regulatory framework for herbal medicines is necessary due to the increased number of unlicensed products on the market. ANVISA has a guide for such studies, called “Guide for the conduction of non-clinical toxicology studies and safety pharmacology required for the development of medicines” (Carvalho et al., 2014).

Information about HDI for the plants to be used by the Brazilian Health system is generally scarce. Those used worldwide, such as *Allium sativum* and *Cynara scolymus* have some interaction data already documented (Mazzari and Prieto, 2014a). Such information is even more scarce for the Brazilian native species, with *Paullinia cupana* as an exception (Rodrigues et al., 2012).

Evidence about HDI is, in most cases, based on alleged relations of the plant constituents with known mechanisms of action. For example, there are no published studies about potential HDI of *Maytenus ilicifolia*. However, it is known that quercetin and kaempferol are *P*-gp modulators found in the plant extract. Therefore, it is assumed but not attested, that HDI are likely to happen in this case (Zhou et al., 2004). As an example, we detected quercetin in our *Lippia sidoides* sample but such amount was not enough to cause changes in *P*-gp activity *in vitro*.

Patients in Brazil do not make exclusive use of registered herbal medicines manufactured by the industry. Local pharmacies are legally allowed to produce pharmaceutical products, which could contain a medicinal plant in the formulation. In this case, the final product does not require the approval of ANVISA to be sold to the patient. Furthermore, medicinal plants are also used in homemade preparations and in those cases data on interactions are even scarcer. ANVISA says that it is time to focus the research on Brazilian native plants in order to reveal the real interactions they could cause. This could improve the knowledge of users, health professionals and even public decision makers.

Experimental studies, such as preclinical studies, which are the subject of this report, are therefore highly important to elucidate underlying mechanisms. Those preclinical studies will serve as guidance for future clinical studies. Eventually, more potential cases of HDI could be prevented rather than being late diagnosed by the pharmacovigilance systems (De Smet, 2007).

## Conclusion

This preclinical study evidences the possibility that the administration of some of these herbal medicines may be able to cause *in vitro* disturbances to metabolic mechanisms. More refined studies would be necessary to ascertain the *in vivo* and/or clinical significance of such interactions. At this point, we can suggest that active pharmacovigilance is recommended for *Cordia verbenaceae, Lippia sidoides*, and especially in the case of *Solanum paniculatum* for which an *in vitro* hPXR-mediated reduction of CYP3A4 gene expression accompanied by significant depletion of glutathione is suggested here for the first time.

Although further studies are needed in order to attest the clinical relevance of our findings, we hope that this work will contribute to stimulate similar research toward the regulation of the quality and safety of these herbal medicines in Brazil.

## Author Contributions

AM contributed to the experimental design and was in charge and performing and setting up all the assays involving Caco-2 and HepG2 cell lines and the writing of the manuscript. SF contributed to HPTLC analysis and to the writing of the manuscript. FdN and FM contributed to the PXR studies. DS contributed by sourcing and extracting the plant material. AC contributed to the regulatory aspects of herbal medicines in Brazil. JP contributed to the experimental design and the writing of the manuscript.

## Conflict of Interest Statement

The authors declare that the research was conducted in the absence of any commercial or financial relationships that could be construed as a potential conflict of interest.

## Footnotes

1 SUS (2009). *Relação Nacional de Plantas Medicinais de Interesse ao SUS (RENISUS)*, Brasilia. Available at: http://bvsms.saude.gov.br/bvs/sus/pdf/marco/ms_relacao_plantas_medicinais_sus_0603.pdf [accessed February 22, 2016].

2 SUS (2010). *Ministério da Saúde. Portaria 886, de 20 de abril de 2010. Institui a Farmácia Viva no âmbito do Sistema Único de Saúde*. Available at: http://bvsms.saude.gov.br/bvs/saudelegis/gm/2010/prt0886_20_04_2010.html [accessed February 22, 2016].
