# Supplementary material for: In vitro Effects of Four Native Brazilian Medicinal Plants in CYP3A4 mRNA Gene Expression, Glutathione Levels, and P-Glycoprotein Activity
Source: Front Pharmacol. 2016 Aug 19;7:265. doi: 10.3389/fphar.2016.00265 (PMC4991120; doi:10.3389/fphar.2016.00265)
Supplement: Supplementary file 1 [file Image_1.PDF]

# *In vitro* effects of four native Brazilian medicinal plants in CYP3A4 mRNA gene expression, glutathione levels and P-glycoprotein activity.

## Supplementary Data

Andre L. D. A. Mazzari[1*], Flora Milton[2], Samantha Frangos[1], Ana Cecília Bezerra Carvalho[3], Dâmaris Silveira[2], Francisco de Assis Rocha Neves[2], Jose M. Prieto[1].

[1] Department of Pharmaceutical and Biological Chemistry, UCL School of Pharmacy, London, UK.

[2] Faculdade de Ciências da Saúde, Universidade de Brasília - Brasília, Distrito Federal, Brazil.

[3] Agência Nacional de Vigilância Sanitária (ANVISA), Coordenação de Medicamentos Fitoterápicos e Dinamizados, Brasília, Distrito Federal, Brazil.

## Table of Contents

**1 Cytotoxicity Studies** .......... 2
*1.1 HeLa cells* .......... 2
*1.2 HepG2 and Caco-2 VCR cells* .......... 2

**2 Real-time qPCR efficiency** .......... 3

**3 Increased efflux activity of P-gp in Caco-2 VCR clone** .......... 5

**4 Effect of the extracts on the plasmids expression in luciferase reporter gene assays** .......... 6
***4.1 Solanum paniculatum displays an antagonistic effect on hPXR luciferase reporter gene assay*** .......... 6
***4.2 Erythrina mulungu displays a partial agonistic effect on hPXR luciferase reporter gene assay*** .......... 7
***4.3 Lippia sidoides does not display any effect on hPXR luciferase reporter gene assay*** 8
***4.4 Cordia verbenaceae displays an antagonistic effect on both hPXR* and $TR_{\beta1}$ but not CMV *luciferase reporter gene assay*** .......... 9

**5 HPTLC ANALYSIS** .......... 11

# 1 CYTOTOXICITY STUDIES

## 1.1 HeLa cells

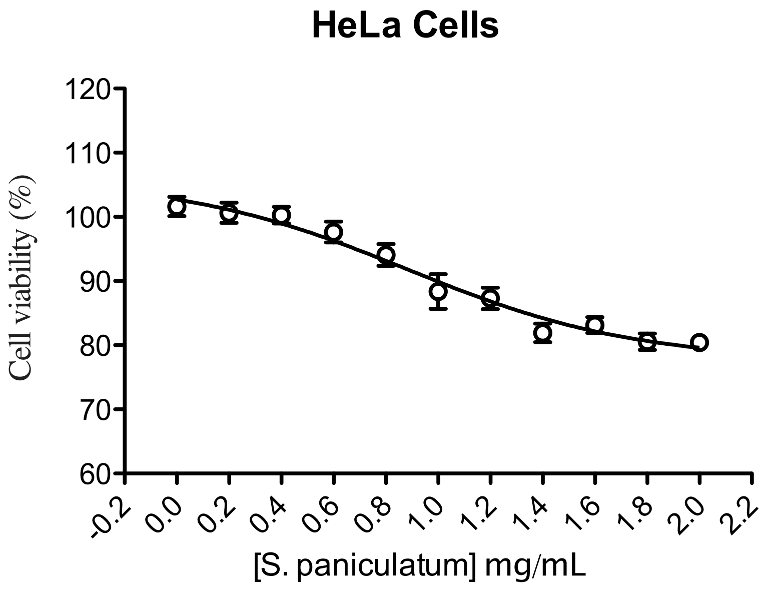


**Figure S 1** HeLa cells were treated with vehicle or increasing concentrations of *S. paniculatum* for 24 hours and then incubated with MTT for additional 4 hours. After complete solubilization of formazan crystals, the absorbance was measured, and cell viability of cells treated with *S. paniculatum* was compared to vehicle (considered 100%).

## 1.2 HepG2 and Caco-2 VCR cells

| Plant extract (100µg/ml) | Viability (%) | |
|---|---|---|
| | HepG2 | Caco-2 VCR |
| *Erythrina mulungu* Benth. | 95.57 ± 0.10 | 97.61 ± 0.17 |
| *Cordia verbenacea* A. DC | 98.16% ± 0.13 | 99.96 ± 0.09 |
| *Solanum paniculatum* L. | 92.47% ± 0.12 | 98.78 ± 0.08 |
| *Lippia sidoides* Cham. | 96.79 ± 0.014 | 96.85 ± 0.05 |

**Table S 1** Cytotoxicity of HepG2 and Caco-2 VCR cells treated with 100µg/ml of the samples for 24h.

# 2 REAL-TIME QPCR EFFICIENCY

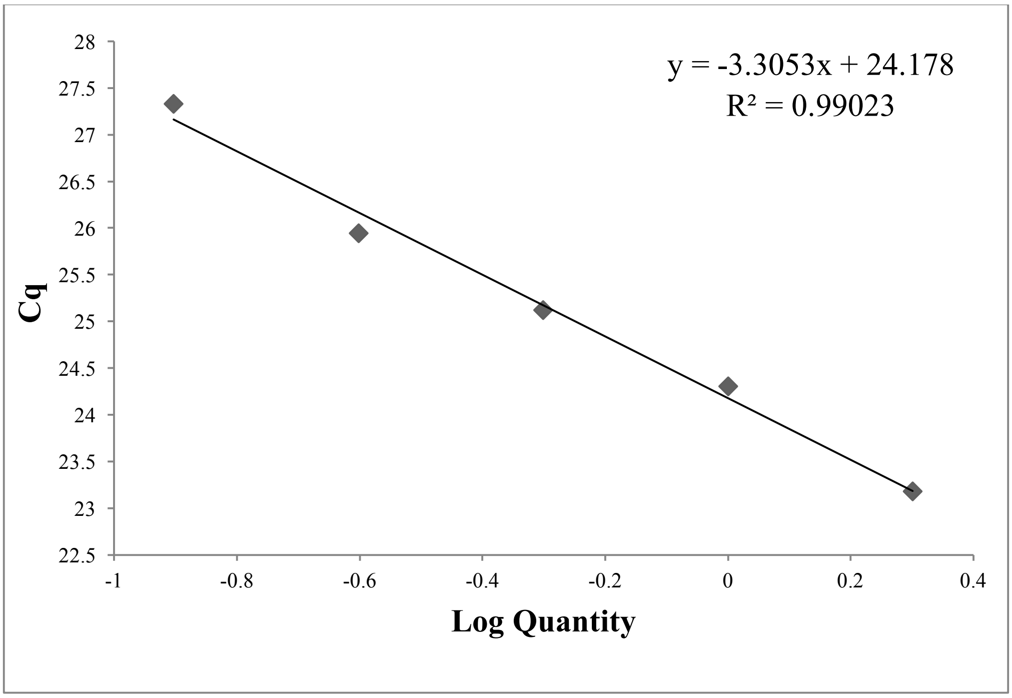


**Figure S 2** CYP3A4 calibration curve.

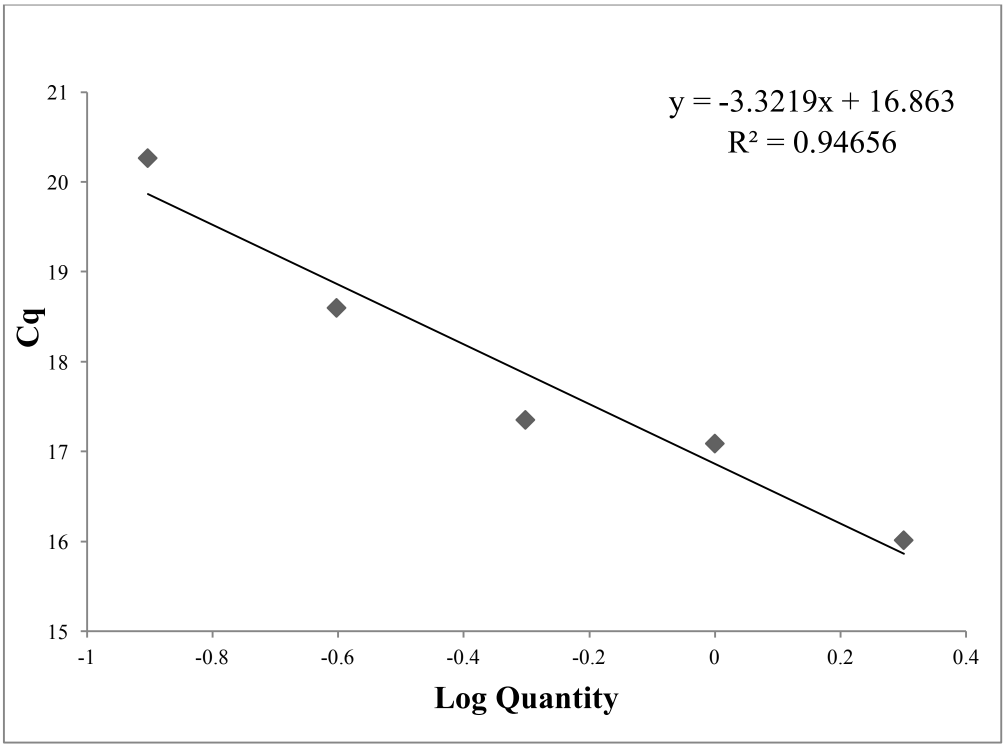


**Figure S 3** β-actin calibration curve.

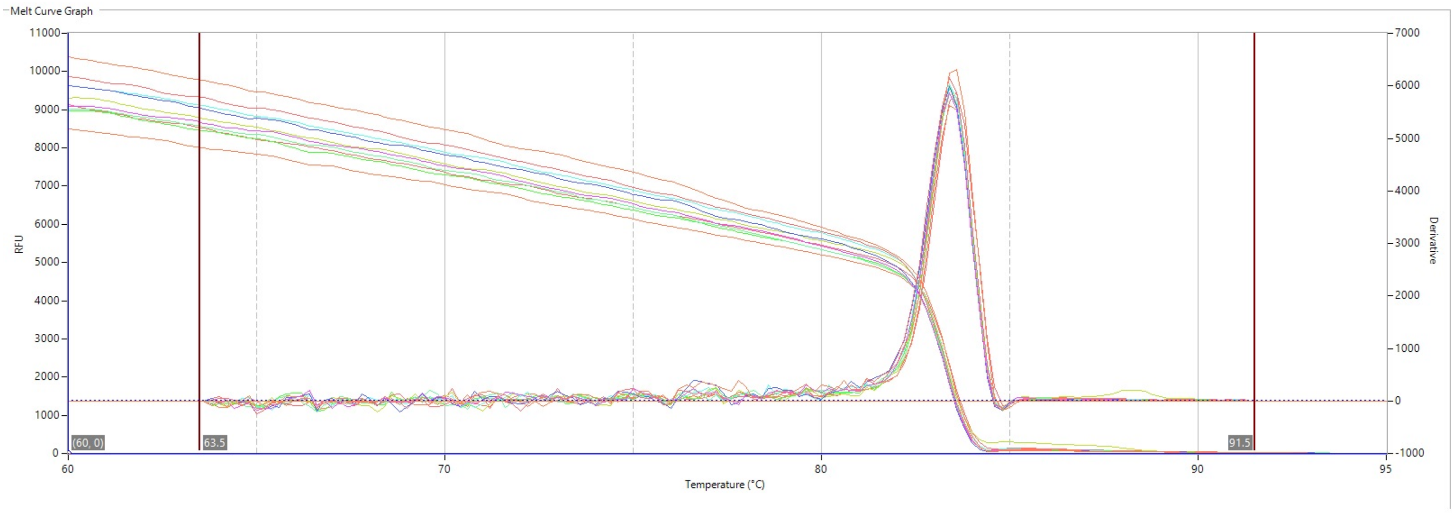


**Figure S 4** CYP3A4 primer melting curve.

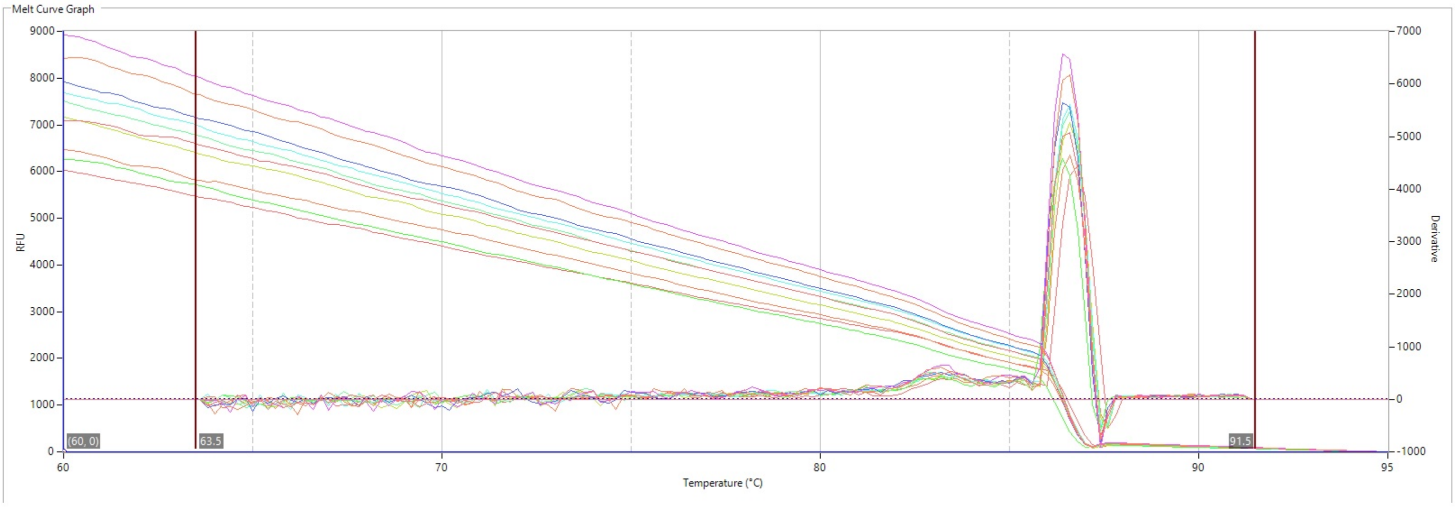


**Figure S 5** β-actin primer melting curve.

# 3 INCREASED EFFLUX ACTIVITY OF P-GP IN CACO-2 VCR CLONE

By culturing wild type Caco-2 cells (Caco-2 WT) in increasing concentrations of vincristine (VCR) over a period of time, P-gp efflux activity can be enhanced in cells with previously low levels of P-gp (Eneroth et al. 2001). The intracellular accumulation of P-gp substrates such as rhodamine-123 (Rh) is inversely proportional to the activity of the transporter in these P-gp expressing cells. Cells with higher P-gp efflux activity typically show lower intracellular concentrations of Rh. Similarly, the addition of a P-gp inhibitor, such as verapamil (VP) will limit its efflux activity with a resultant increase in intracellular concentration of the P-gp substrate.

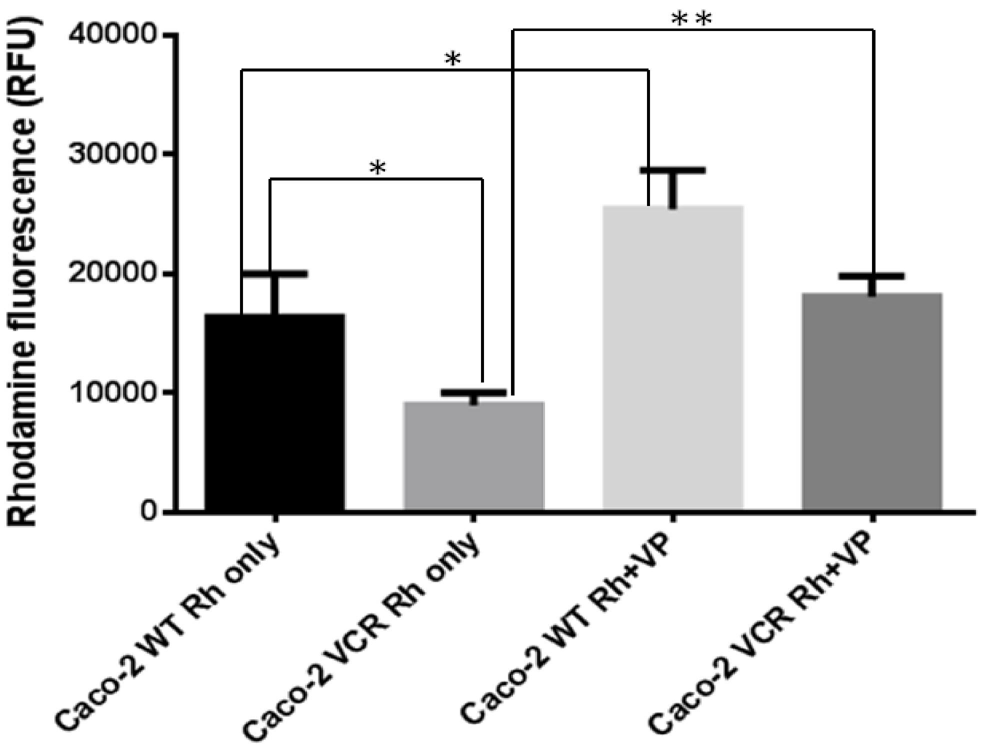


**Figure S 6** P-gp efflux activity of wild type Caco-2 (Caco-2 WT) and vincristine resistant Caco-2 (Caco-2 VCR) cells in presence or not of the P-gp inhibitor verapamil (VP) in cell culture media containing rhodamine-123 (Rh). Detection of rhodamine fluorescence (RFU) indicates the level of P-gp efflux activity in the cell lines. Results show mean ± S.D, n=3. Statistical significance based on a paired t-test. *$p \leq 0.05$; **$p \leq 0.01$.

To confirm this, we measured the intracellular Rh fluorescence in both Caco-2 WT and vincristine resistant Caco-2 (Caco-2 VCR) cells incubated for two hours with 5µg/ml of Rh in the presence or absence of 20µM VP. As shown in figure S6, the intracellular Rh fluorescence measured in relative fluorescence units (RFU) was lower in the Caco-2 VCR cells compared to Caco-2 WT cells. Addition of VP brought about an increase in intracellular RFU in both the Caco-2 VCR and the Caco-2 WT cells, with the first being always significant lower than the latter.

# 4 EFFECT OF THE EXTRACTS ON THE PLASMIDS EXPRESSION IN LUCIFERASE REPORTER GENE ASSAYS

## 4.1 *Solanum paniculatum displays an antagonistic effect on hPXR luciferase reporter gene assay.*

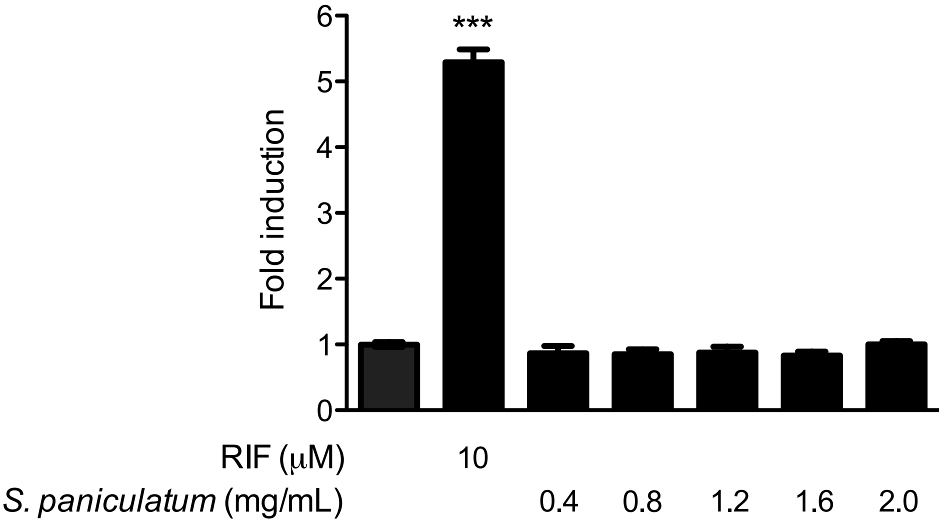


**Figure S 7 Agonistic effect assay.** HeLa cells were co-transfected with expression vector pM-Gal4-PXR-LBD and Gal4 luciferase reporter and treated with vehicle, rifampicin (RIF) or increasing concentrations of *S. paniculatum*. Luciferase activity was measured after 24 hours and reported as fold induction compared to vehicle. Results represent data from three independent experiments performed in triplicate. *** $p \leq 0.001$.

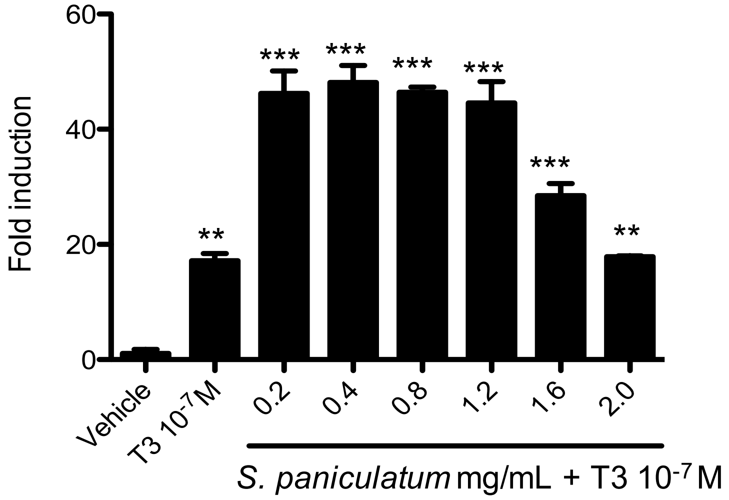


**Figure S 8** HeLa cells were co-transfected with expression vector pM-Gal4-dTR$_{\beta 1}$-LBD and Gal4 luciferase reporter and treated with vehicle, T3 or increasing concentrations of *S. paniculatum*. Luciferase activity was measured after 24 hours and reported as fold induction compared to vehicle. **$p \leq 0.01$; ***$p \leq 0.001$.

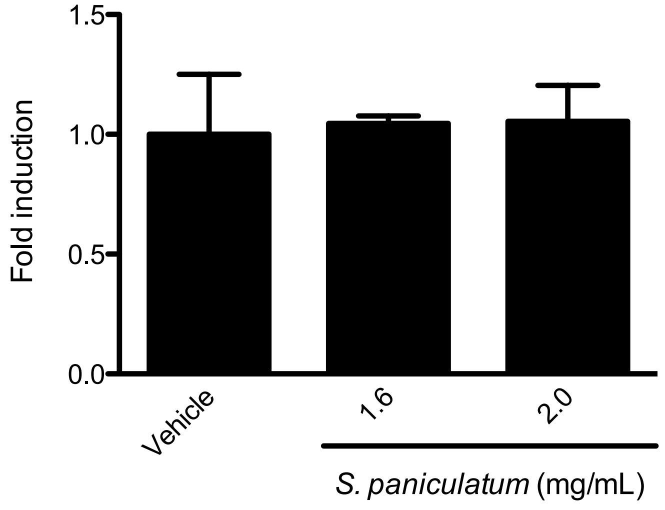


**Figure S 9** HeLa cells were transfected with expression vector CMV luciferase reporter and treated with vehicle, 1.6 or 2.0 mg/ mL of *S. paniculatum*. Luciferase activity was measured after 24 hours and reported as fold induction compared to vehicle.

## *4.2 Erythrina mulungu displays a partial agonistic effect on hPXR luciferase reporter gene assay.*

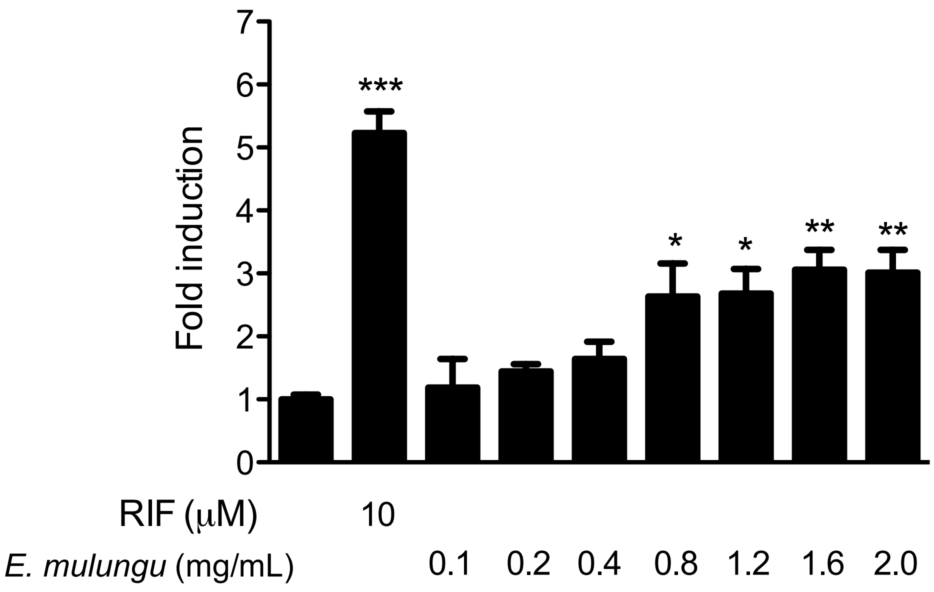


**Figure S 10 Agonistic effect assay.** HeLa cells were co-transfected with expression vector pM-Gal4-PXR-LBD and Gal4 luciferase reporter and treated with vehicle, rifampicin (RIF) or increasing concentrations of *E. mulungu*. Luciferase activity was measured after 24 hours and reported as fold induction compared to vehicle. *p ≤0.05; **p ≤ 0.01; ***p ≤ 0.001.

## 4.3 *Lippia sidoides does not display any effect on hPXR luciferase reporter gene assay.*

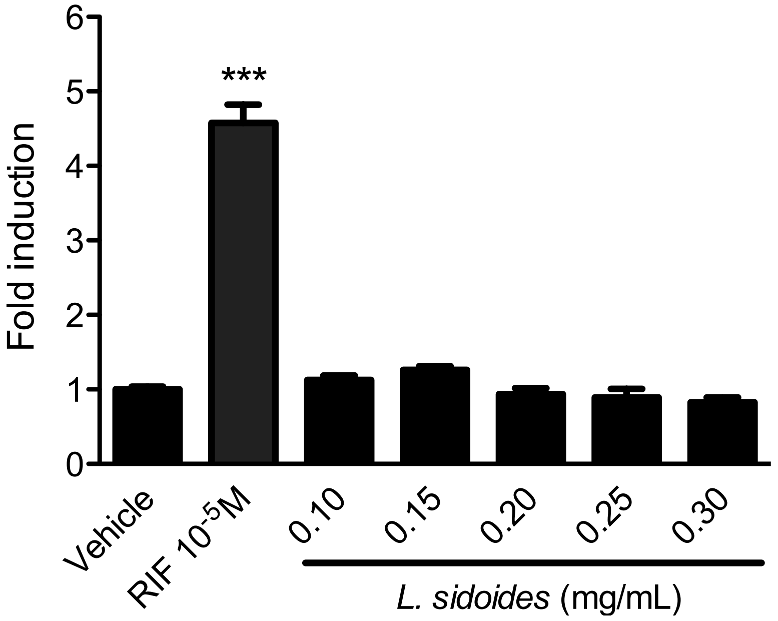


**Figure S 11 Agonistic effect assay.** HeLa cells were co-transfected with expression vector pM-Gal4-PXR-LBD and Gal4 luciferase reporter and treated with vehicle, rifampicin (RIF) or increasing concentrations of *L. sidoides*. Luciferase activity was measured after 24 hours and reported as fold induction compared to vehicle. ***$p \leq 0.001$.

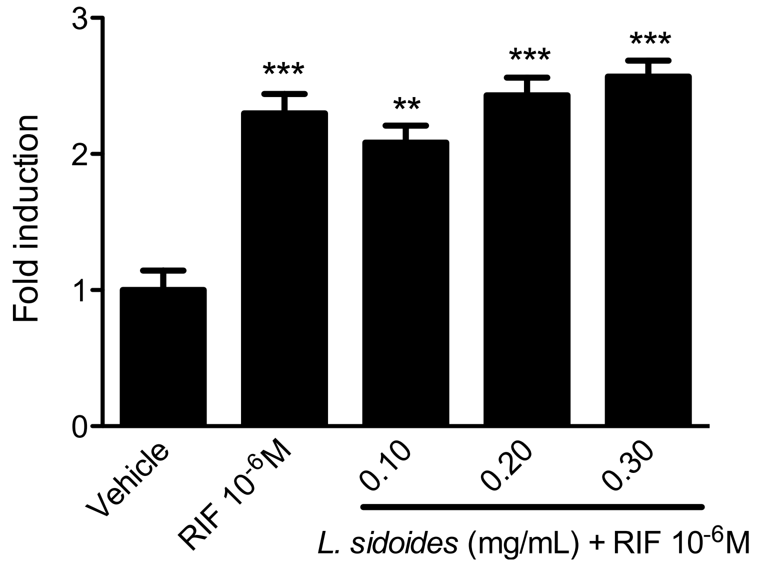


**Figure S 12 Antagonistic effect assay.** HeLa cells were co-transfected with expression vector pM-Gal4-PXR-LBD and Gal4 luciferase reporter and treated with vehicle, rifampicin (RIF) without and with increasing concentrations of *L. sidoides*. Luciferase activity was measured after 24 hours and reported as fold induction compared to vehicle. **$p \leq 0.01$. ***$p \leq 0.001$.

## *4.4 Cordia verbenaceae displays an antagonistic effect on both hPXR and $TR_{\beta 1}$ but not CMV luciferase reporter gene assay.*

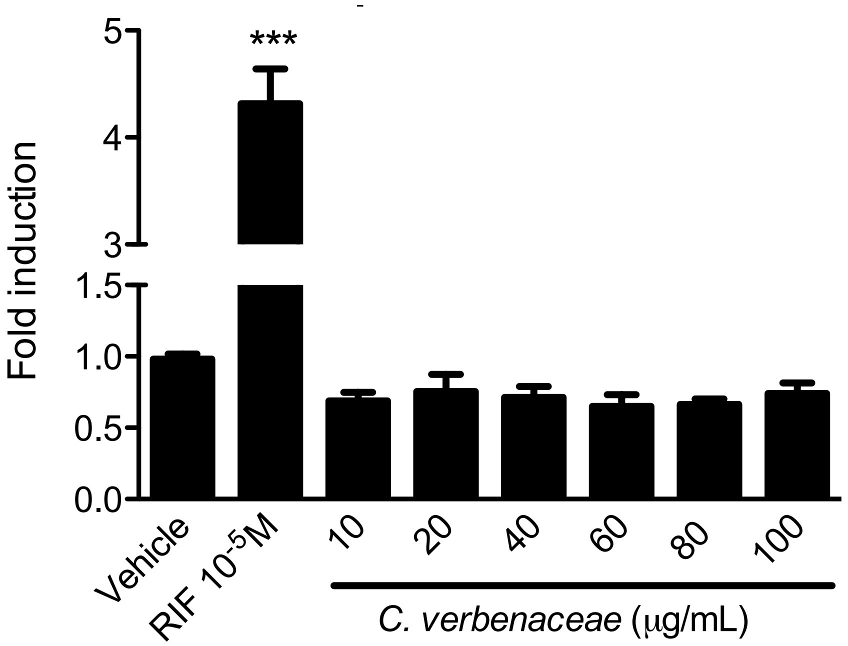


**Figure S 13 Agonistic effect assay.** HeLa cells were co-transfected with expression vector pM-Gal4-PXR-LBD and Gal4 luciferase reporter and treated with vehicle, rifampicin (RIF) or increasing concentrations of *C. verbenaceae*. Luciferase activity was measured after 24 hours and reported as fold induction compared to vehicle. ***$p \leq 0.001$.

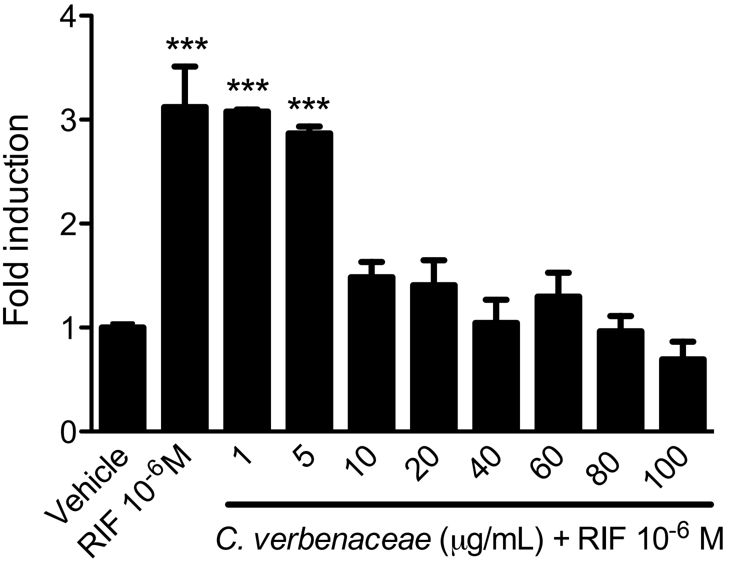


**Figure S 14 Antagonistic effect assay.** HeLa cells were co-transfected with expression vector pM-Gal4-PXR-LBD and Gal4 luciferase reporter and treated with vehicle, rifampicin (RIF) without or with increasing concentrations of *C. verbenaceae*. Luciferase activity was measured after 24 hours and reported as fold induction compared to vehicle. ***$p \leq 0.001$.

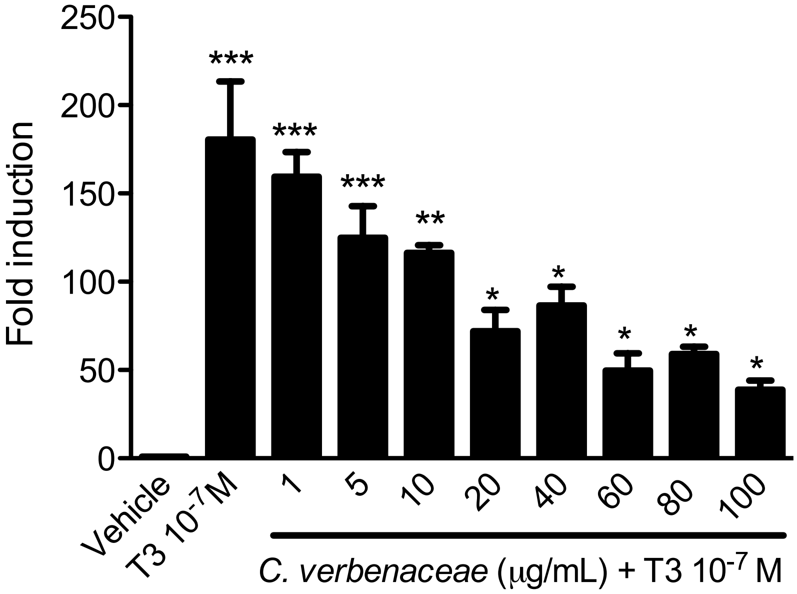


**Figure S 15 Antagonistic effect assay on $TR_{\beta1}$.** HeLa cells were co-transfected with expression vector pM-Gal4-d$TR_{\beta1}$-LBD and Gal4 luciferase reporter and treated with vehicle, T3 without and with increasing concentrations of *C. verbenaceae*. Luciferase activity was measured after 24 hours and reported as fold induction compared to vehicle. *$p \leq 0.05$; **$p \leq 0.01$; ***$p \leq 0.001$.

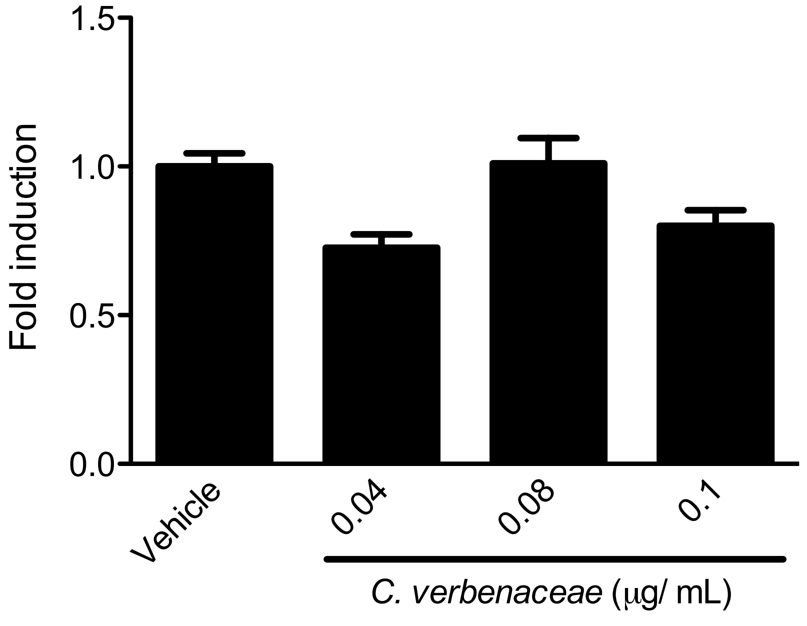


**Figure S 16** HeLa cells were co-transfected with expression vector CMV luciferase reporter and treated with vehicle or with increasing concentrations of *C. verbenaceae*. Luciferase activity was measured after 24 hours and reported as fold induction compared to vehicle.

# 5 HPTLC ANALYSIS

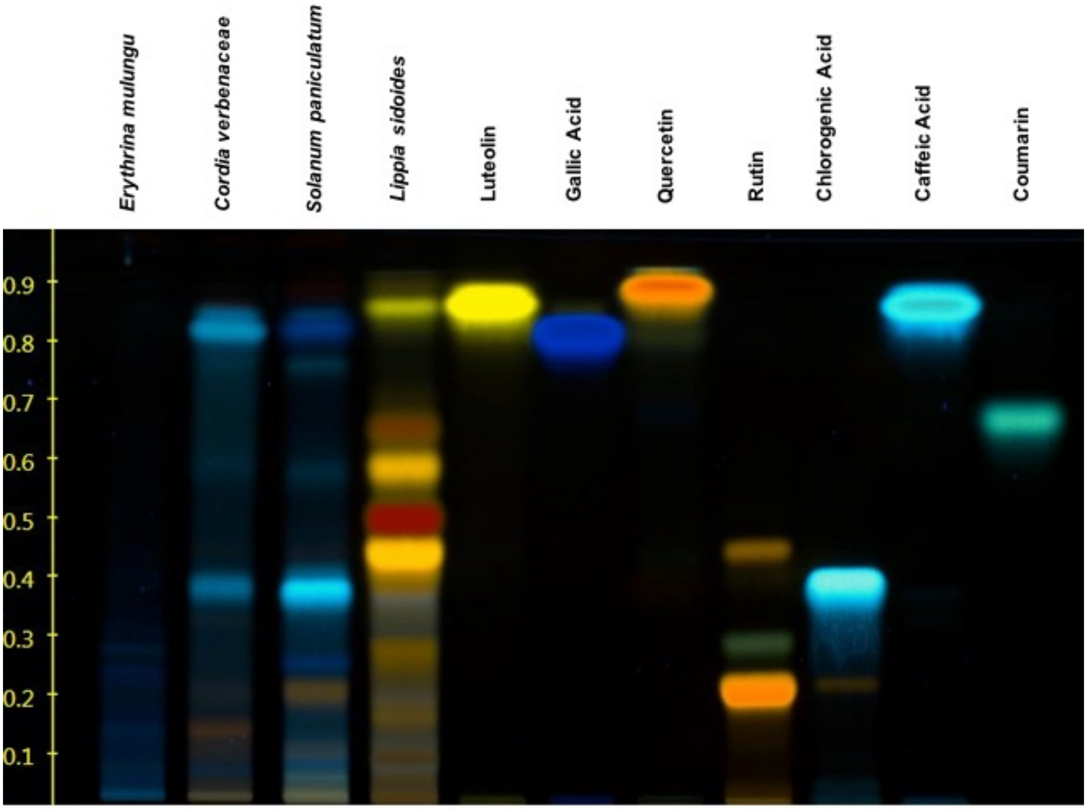


**Figure S 17** 365nm after derivatization

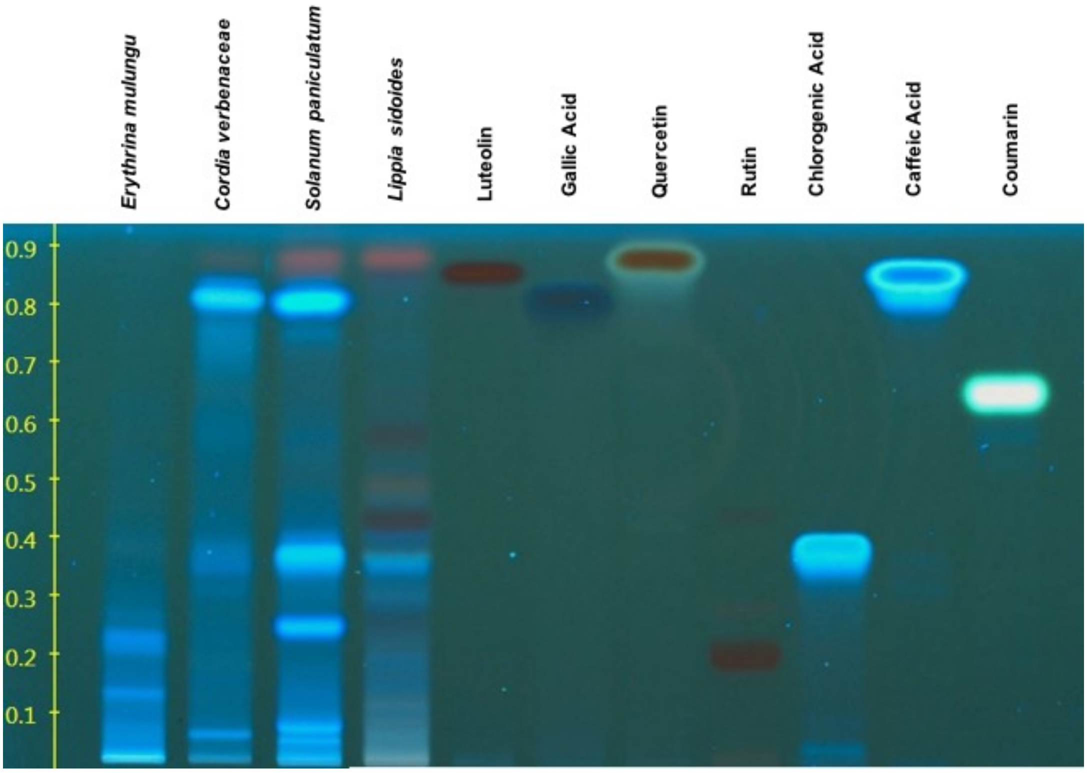


**Figure S 18** 365nm before derivatization

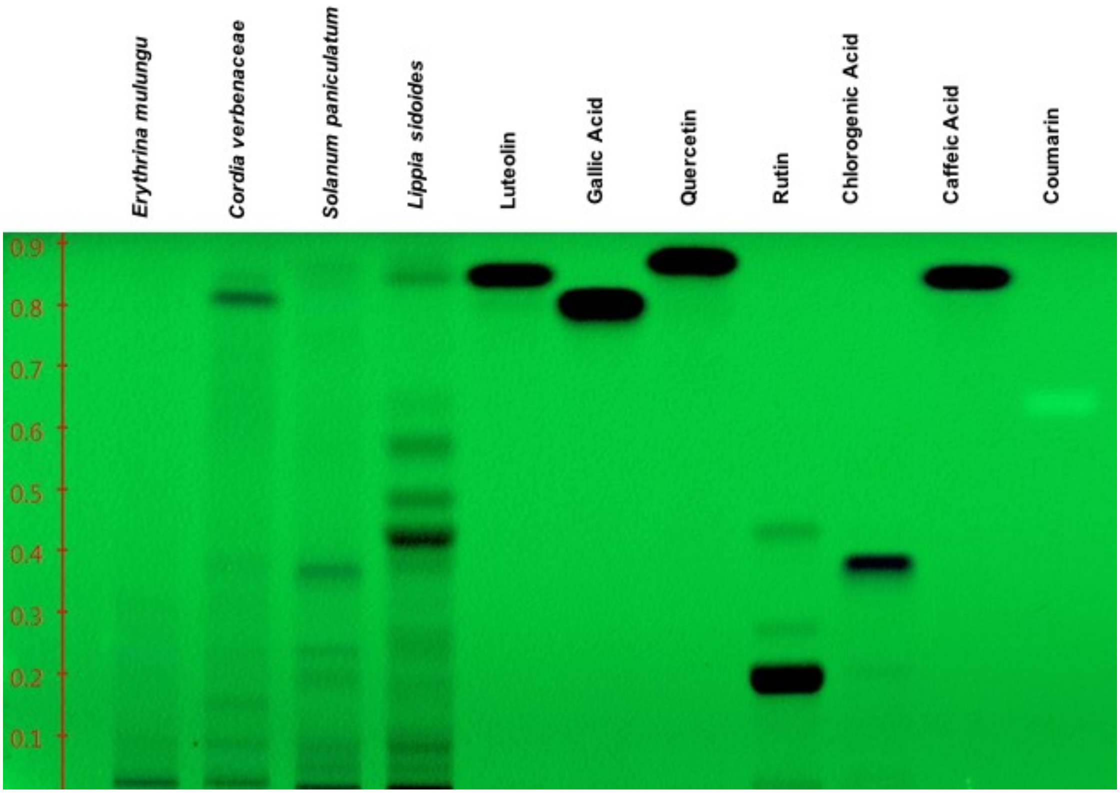


**Figure S 19** 235 nm after derivatization
